# A Novel Cell-Based Hybrid Acoustic Wave Biosensor with Impedimetric Sensing Capabilities

**DOI:** 10.3390/s130303039

**Published:** 2013-03-04

**Authors:** Fei Liu, Fang Li, Anis Nurashikin Nordin, Ioana Voiculescu

**Affiliations:** 1 Mechanical Engineering Department, City College of New York, New York, NY 10031, USA; E-Mail: fliu02@ccny.cuny.edu; 2 Mechanical Engineering Department, New York Institute of Technology, Old Westbury, NY 11568, USA; E-Mail: fangli77@gmail.com; 3 Electrical and Computer Engineering, International Islamic University Malaysia, Jalan Gombak, Kuala Lumpur 53100, Malaysia; E-Mail: anis.nordin@gmail.com

**Keywords:** quartz crystal microbalance, thickness shear mode, impedance spectroscopy measurements, resonant frequency, bovine aortic endothelial cells, COMSOL Multiphysics™

## Abstract

A novel multiparametric biosensor system based on living cells will be presented. The biosensor system includes two biosensing techniques on a single device: resonant frequency measurements and electric cell-substrate impedance sensing (ECIS). The multiparametric sensor system is based on the innovative use of the upper electrode of a quartz crystal microbalance (QCM) resonator as working electrode for the ECIS technique. The QCM acoustic wave sensor consists of a thin AT-cut quartz substrate with two gold electrodes on opposite sides. For integration of the QCM with the ECIS technique a semicircular counter electrode was fabricated near the upper electrode on the same side of the quartz crystal. Bovine aortic endothelial live cells (BAECs) were successfully cultured on this hybrid biosensor. Finite element modeling of the bulk acoustic wave resonator using COMSOL simulations was performed. Simultaneous gravimetric and impedimetric measurements performed over a period of time on the same cell culture were conducted to validate the device's sensitivity. The time necessary for the BAEC cells to attach and form a compact monolayer on the biosensor was 35∼45 minutes for 1.5 × 10^4^ cells/cm^2^ BAECs; 60 minutes for 2.0 × 10^4^ cells/cm^2^ BAECs; 70 minutes for 3.0 × 10^4^ cells/cm^2^ BAECs; and 100 minutes for 5.0 × 10^4^ cells/cm^2^ BAECs. It was demonstrated that this time is the same for both gravimetric and impedimetric measurements. This hybrid biosensor will be employed in the future for water toxicity detection.

## 1. Introduction

Cell-based biosensor systems that incorporate whole cells have the capacity to respond to a wide range of analytes in a physiologically relevant manner [1]. By employing living cells as sensors, bioanalytes can be screened without requiring *a priori* knowledge of the analyte's chemistry. Cell-based assays are emerging as a promising approach to monitor the presence of pathogens in clinical, environmental, or food samples or to conduct cytotoxic testing of drugs and toxicants [2–6]. Living cells are extremely sensitive to modulations or disturbances in physiological microenvironment. Therefore, they could be also employed to screen pharmaceutical drugs or environmental agents capable of causing perturbations or apoptosis of cells [5,6]. Cell-based biosensors monitor physiological changes in reporter cells exposed to a wide range of analytes including pathogens, chemical pollutants, toxic industrial chemicals, biomolecules, or drugs. Live cells actively define the transduction mechanism and toxin detection is based solely on physiological cell responses, with cell death being an indicator of a potential threat to human health.

Electro-acoustic sensors are employed in a wide range of applications due to their high sensitivity and wireless capabilities. These sensors have high frequency (MHz–GHz) acoustic waves travelling through or on top of a piezoelectric substrate. The acoustic waves are sensitive to any change both on the surface and in the piezoelectric material. Variations such as mass loading or viscosity, that occurs in the propagation path of the acoustic waves, cause their velocity and amplitude to change [7]. These acoustic wave sensors could be employed to monitor physiological changes in reporter cells exposed to a wide range of analytes. The cell's biophysical properties such as adhesion strength, as well as minute alterations of cell mass can be detected by measuring the shift in the resonant frequency and the insertion loss of the acoustic wave [8]. Few types of acoustic wave devices could be integrated in microfluidic systems and seeded with live cells without significant degradation of the quality factor. The Quartz Crystal Microbalance (QCM) is an extremely sensitive mass sensor, capable of measuring mass changes in the nanogram range. The QCM is a piezoelectric thickness-shear-mode resonator that has been successfully employed for physical, chemical and biological sensing applications due to the minimal damping of the acoustic wave [9–11]. Moreover, QCMs could be adapted for many different applications by developing coatings that respond to different target molecules, adding to their versatility. QCM resonators have been used to detect the adhesion of a cell monolayer based on monitoring the resonance frequency shifts [12,13]. The cells were considered as viscoelastic material and the viscoelastic properties of cell monolayer were also characterized using QCM resonators [14–16].

The QCM could be combined with electrochemical impedance spectroscopy (EIS) to provide complementary information about biological processes formed on the upper electrode. The combination of EIS and QCM was used for simultaneous monitoring of interaction of bovine serum albumin (BSA) protein with copper [17]. For this application a commercial QCM was used and copper films were electroplated onto the upper gold electrode of the commercial QCM. The QCM was introduced in an electrochemical cell containing a large Pt foil counter electrode and a saturated calomel reference electrode. The upper gold electrode of the QCM was considered the working electrode. Experiments were conducted in the presence and absence of BSA and the results showed specific interactions between BSA and copper. Commercial QCM combined with EIS technique was also used for the study of DNA hybridization that was simultaneously detected by EIS and QCM experiments [18]. For performing EIS, the upper QCM platinum excitation electrode was used as working electrode, the counter electrode was a platinum wire and a silver wire was used as reference electrode. Electrochemical impedance spectroscopy experiments were performed for direct detection of DNA hybridization and the results were compared to quartz crystal microbalance measurements. Recently, combined QCM and EIS technique was used for the study of supported lipid bilayer (SLB) formation and interaction with pore-forming peptides [19]. In this study the upper electrode of a commercial QCM was combined with Pt foil counter electrode and Ag/AgCl reference electrode for performing EIS measurements. Supported lipid bilayers were assembled on the QCM upper electrode and the formation of pores from membrane-inserting peptides was observed with both techniques: mass variations and EIS measurements. The combined setup has emphasized phenomena regarding both; the mechanism of SLB formation and the effects of pore-forming peptide insertion on SLB properties, which could not have been detected using only one of the techniques. The combination of QCM with EIS provides complementary information about the biological system under study.

This paper presents the design and fabrication of a hybrid cell-based sensor that integrates both; acoustic wave sensing and electrochemical sensing in a novel device. The existing combinations of QCM and EIS reported in the literature have large dimensions; the working electrode diameter is 5 mm and the dimensions of counter and reference electrodes are in centimeters [17–19]. In contrast, the biosensor presented in this paper includes a miniature QCM, with electrode diameter of 2 mm, and an electric cell-substrate impedance sensing (ECIS) [20–35] system fabricated on a quartz substrate. The ECIS semicircular counter electrode is fabricated around the upper working electrode on the same side of the quartz crystal.

The innovative aspects of the presented research are: (1) the fabrication of a counter ECIS electrode with miniature dimensions on the quartz substrate on the same side with the QCM working electrode and (2) the combination of QCM and ECIS is used for the first time to study live mammalian cells.

There are several possible applications of this original multiparametric sensor system such as: pharmacology, cell biology, toxicology, and environmental monitoring. In all these application live mammalian cells cultured on the biosensor will be exposed to pharmacological drugs or environmental toxicants. Based on the results obtained from the QCM and ECIS measurements the viability of the cells affected by the toxicants will be assessed.

Recently, there has been interest in developing biosensors based on live mammalian cells to rapidly monitor the toxicity of water samples [36,37]. These cell based sensors use living cells as sensorial elements to monitor water toxicity. In this research the cell's viability after exposure to toxic water is monitored only by electric cell-substrate impedance (ECIS) technique. When cells attach and spread on the surface of planar ECIS electrodes, the cells membrane have dielectric properties and the current is constrained to flow through narrow gaps between cells and working electrode surface. Measurements of the electrical impedance of the cell-covered electrode contain information about the dynamics of cells attachment, cells movements and viability.

The novel device presented in this paper will have higher accuracy of detection, compared to a sensor with only a single sensing method [36,37]. When the cells cultured on this hybrid sensor are exposed to toxicant and are apoptotic the impedance values are minimal since the cells lose their dielectric properties. Apoptotic cells also become detached from the QCM electrode and the resonant frequency increases. For rapid studies of water toxicity the simultaneous response from two different sensors will confirm the quality of the water. In this paper Bovine aortic endothelial cells (BAEC) will be cultured on the hybrid sensor. Resonant frequency shifts and ECIS measurements due to cell attachment and growth will also be presented. This research demonstrated that an acoustic wave resonator can be successfully integrated with an electrochemical sensor on a quartz substrate. BAEC cells (VEC Technology) can be cultured on the hybrid sensor and precise electric measurements could be recorded.

## 2. Device Concept and Operation

**Figure 1.**
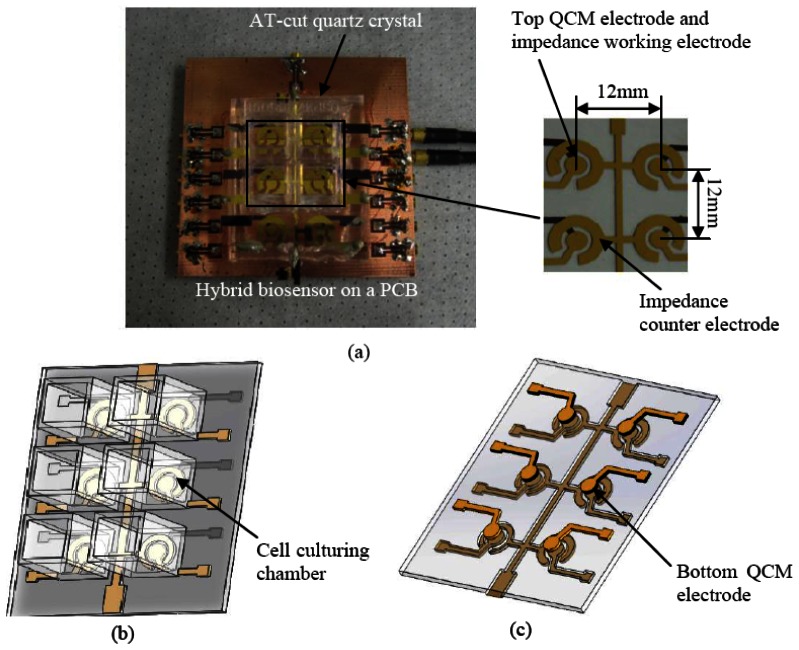
The hybrid sensors configuration (2 × 3 array). (**a**). The image of the hybrid sensor array on PCB; (**b**). Top view of the sensor array assembled with cells culturing chamber; (**c**). Bottom view of the sensor showing bottom QCM electrode.

The sensor is based on the innovative employment of the upper QCM electrode as the working electrode for ECIS technique as shown in Figure 1. The QCM acoustic wave sensor consists of a thin AT-cut quartz substrate with two electrodes on opposite sides. The ECIS semicircular counter electrode was fabricated near the QCM upper electrode on the same side of the quartz substrate.

**Figure 2.**
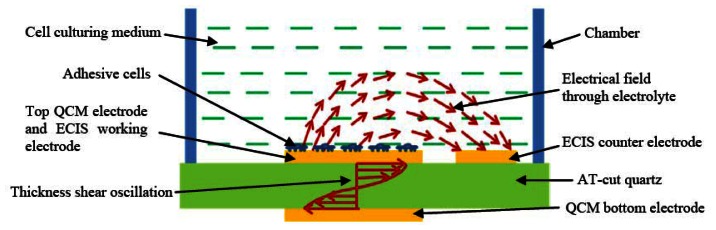
Illustration of the working principle of the hybrid biosensor which integrates acoustic wave sensing with impedance spectroscopy techniques.

The schematic of the hybrid sensor working principle is illustrated in Figure 2. As illustrated in Figure 2, an alternating current applied between the top and bottom QCM electrodes generates thickness shear mode acoustic waves that propagate through the quartz substrate. The resonant frequency of the QCM could be calculated with Equation (1) [38]:

(1) $$f_{0}=\sqrt{\frac{\mu_{q}}{\rho_{q}}/2t_{q}}$$

where t_q_ is the quartz crystal's thickness, ρ_q_ is the quartz density and μ_q_ is the shear modulus.

Based on Equation (1) it can be seen that if the density of the QCM changes, the resonant frequency of the device also changes, making the QCM suitable for monitoring changes in mass. In the case of this research, the mammalian cells will be cultured on the combination of QCM and ECIS electrodes, which are covered with a layer of extracellular matrix (ECM) required to improve the mammalian cell attachment to the device. When the mammalian cells attach to QCM its resonant frequency decreases. In contrast, when the mammalian cells detach from the substrate its resonant frequency increases. When the cells are affected by drugs or toxicants they undergo apoptosis and their attachment to the QCM become less strong and eventually the apoptotic cells detach from the QCM. Information about cell attachment and viability could be obtained by monitoring the QCM resonance frequency shifts.

The device presented in Figure 1(a) could simultaneously perform resonant frequency measurements and impedance measurements on the same cell monolayer cultured on the QCM upper electrode, which is also the working electrode of the ECIS system. When alternating current is applied on ECIS working and circular counter electrodes, an electrical field is generated through the cell culture medium, as seen in Figure 2. The electrical impedance between these electrodes could be recorded over a wide frequency range (40 Hz to 100 kHz) as a function of time. The amplitude of current passing through the cell is very low, in the nanoampere (nA) range. This low current creates a negligible electrical stimulation to the cell during the impedance measurement and the cells viability is not affected. The existence of membrane potential is a distinguishing feature between living and non-living cells. Impedance measurements of cells can differentiate between normal and abnormal cell types. Healthy cells adhere more tightly to a surface in comparison to unhealthy or dead cells. When cells attach and spread on the surface of these planar electrodes for ECIS measurements because the dielectric properties of cell membrane the current is constraint to flow through narrow gaps between cells into the cell media, which acts as an electrolyte. Measurements of the electrical impedance of the cell-covered electrode contain information about the cell attachment, shape and viability. Upon the attachment of cells on the electrodes, the impedance increases because the cells act as insulating particles restricting the current flow. When the cells are apoptotic as a result of contamination or exposure to toxicants or drugs the cell impedance will abruptly decrease because the cell membrane loses its dielectric properties.

## 3. Materials and Methods

**Figure 3.**
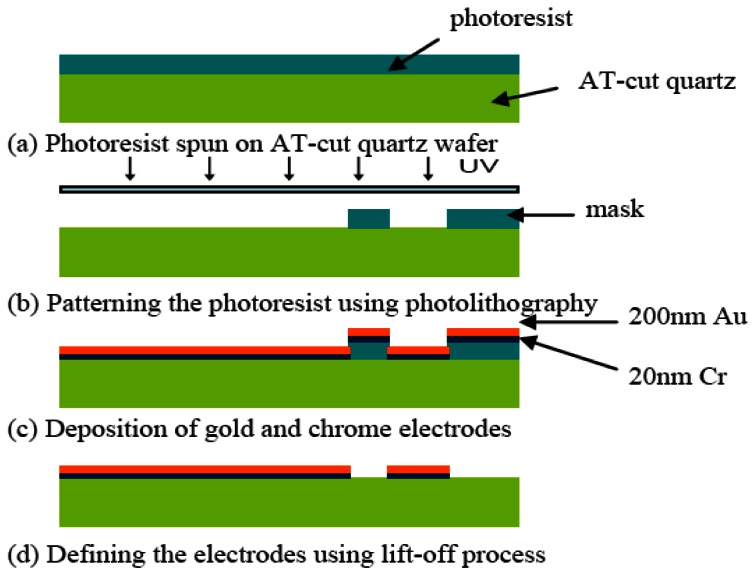
Illustration of the microfabrication process flow of the hybrid biosensor fabricated on an AT-cut quartz substrate.

The hybrid sensor was fabricated using microfabrication process. The fabrication flow is illustrated in Figure 3. A 20 nm chrome (Cr) layer and 200 nm of gold (Au) layer were deposited using thermal evaporation on the front side and back side of an AT-cut quartz substrate with a nominal thickness of 100 μm. The Cr layer is necessary for increasing the adhesion of the Au layer on the quartz substrate. The circular QCM electrodes and ECIS counter electrode were patterned using photolithography. The QCM top and bottom electrodes have a diameter of 2 mm. An array of six identical hybrid biosensors with high-throughput were fabricated on the quartz substrate as illustrated in Figures 1. The center to center distance of the adjacent hybrid biosensors is 12 mm. This distance allows minimization of the signal interference between different channels [39].

## 4. Simulation and Experimental Characterization of the Hybrid Biosensor

### 4.1. Electric Characterization without Cells

**Figure 4.**
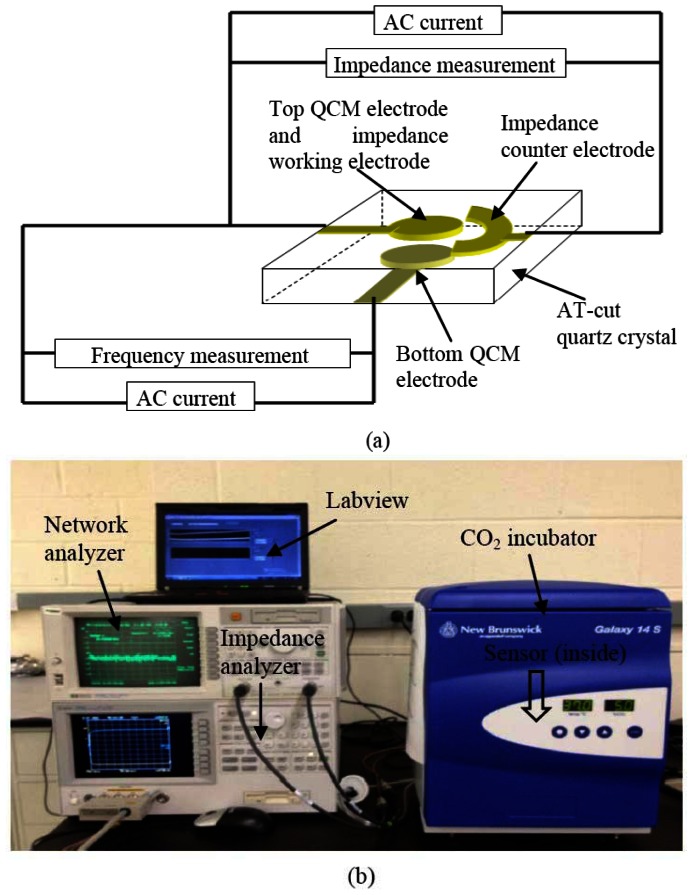
(**a**) The schematic of experimental testing structure of the hybrid biosensor. (**b**) The image of the experimental setup. The biosensor is located in the CO_2_ incubator, and connected with instruments for measurements.

The schematic of experimental configuration and measurement setup are illustrated in Figure 4(a) and (b). Each hybrid biosensor was enclosed in a commercial polystyrene cell culturing chamber from Thermo Scientific (New York, NY, USA). The commercial culturing chambers were bonded on the quartz substrate using silicone glue. To minimize the damping effect of the quartz substrate, only the edges of the rectangular quartz substrate were bonded on a printed circuit board (PCB) using a PDMS membrane, that allows the central part of the rectangular quartz substrate to be suspended. The PCB with subminiature version A (SMA) connectors, are used to interface with the network analyzer (Agilent HP-8714ET) and impedance analyzer (Agilent 4294A), and were connected with a computer via general purpose interface bus (GPIB). A program was created on computer to acquire data using Labview 8.5 from National Instrument (Austin, TX, USA), as seen in Figure 4(b).

To monitor the cells' growth, the whole sensor was placed inside a humidified incubator with 5% CO_2_ at 37 °C. A large polystyrene lid from Thermo Scientific was used to cover the six culturing chambers of the hybrid biosensor array to prevent the evaporation of culture medium. Prior to the experiment, the chip was washed with phosphate buffered saline (PBS) and distilled water three times, followed by exposure to UV light for 10 minutes. The lid was sterilized with ethanol.

Finite element simulation using COMSOL Multiphysics™ (Burlington, MA, USA) a commercially available modelling package, was performed to model the resonant frequency of the QCM resonator and thickness shear deformation. The resonator was modeled in three dimensions (3D), as an AT-cut quartz substrate of 100 μm thickness. The material properties of the AT-cut quartz crystal substrate are shown in Table 1. The top and bottom circular excitation electrodes were modeled as gold (Au) films of 200 nm thickness. The 3D model was meshed using the automatic mesher. A sinusoidal voltage with amplitude of 5 mV was applied across the quartz crystal. The eigenfrequency analysis was performed to yield the quartz surface displacement and thickness shear deformation. The frequency domain analysis was conducted to obtain the resonant frequency of the resonator.

**Figure 5.**
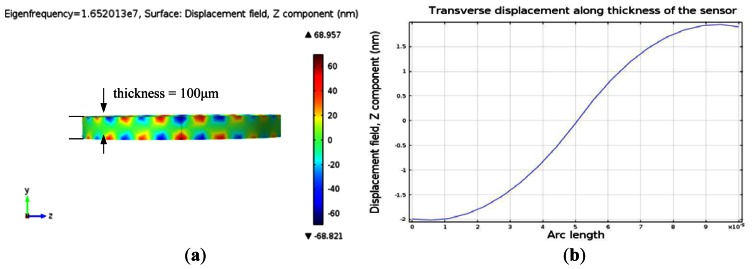
COMSOL simulation of the acoustic wave gravimetric sensor. (**a**) 3D model and surface displacement field at resonant frequency of 16.52 MHz. (**b**) Transverse displacement along thickness of the AT-cut quartz substrate.

**Figure 6.**
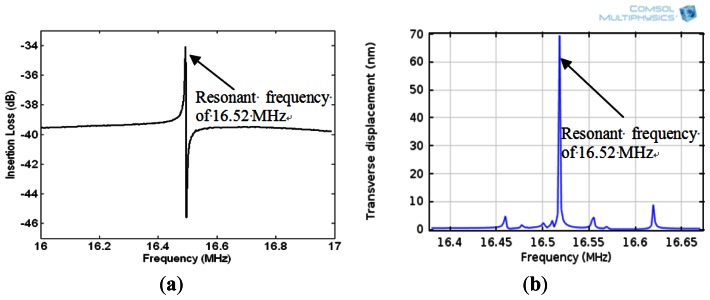
(**a**) The insertion loss of the sensor measured using network analyzer which shows the resonant frequency at 16.52 MHz. (**b**) The COMSOL simulation of the acoustic wave sensor's transverse displacement with maximum displacement also at 16.52 MHz.

Figure 5(a) illustrates the eigenfrequency of the QCM and the displacement at the surface at resonance. The transverse displacement along the thickness of the quartz substrate at resonance is shown in Figure 5(b). Figure 5(b) indicates that a standing wave is formed along the thickness of the quartz substrate at resonant frequency with half wave length equalling the quartz thickness. Moreover, the shear deformation is zero at half of the quartz thickness and becomes maximum with opposite phase at the top and bottom surfaces of the quartz substrate at resonance. The resonant frequency of the device is the frequency which generates maximum displacement in the z-domain and it is equal to 16.52 MHz (Figure 6(b)). The insertion loss of the sensor was measured with network analyzer (Figure 6(a)) and showed a good match of the resonant frequency with the simulated model as seen in (Figure 6(b)).

**Figure 7.**
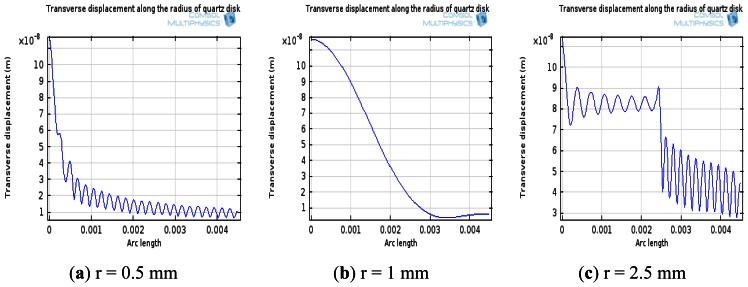
The simulation results of transverse displacements along the radius of AT-cut quartz disk with different sizes of electrodes.

The simulation of transverse displacements of AT-cut quartz with different electrode sizes at resonance was performed as seen in Figure 7. In this simulation, the electrode radius was modified from 0.5 mm to 2.5 mm. From Figure 7, it was found that the value of quality factor presents the tendency to increase first and decrease afterwards when the radius of electrode changes from 0.5 mm to 2.5 mm, and the vibration energy can be limited in the electrode area at r = 1 mm. Considering the prediction from the simulation, the electrode diameter was chosen 2 mm.

### 4.2. Electric Characterization with Cells

This hybrid cell-based biosensor was designed to test the toxicity of water. BAECs are the primary cell type used in this toxicity device, because previous studies have determined that they are sensitive to a range of environmental toxicants and exhibit long-term survival without need for laboratory manipulation. These cells could survive for up to six weeks on the fluidic biochips and remain responsive to toxicants. The BAECs were maintained in MCDB-131 complete medium VEC Technology, (Greenville, PA, USA) in a 5% CO_2_ incubator. For experiments with the hybrid biosensor, the confluent BAECs were trypsinized, followed by resuspension in culture medium. Cell counting with a hemocytometer was used to prepare a specific concentration of cells. As extracellular matrix (ECM) 100 μL of fibronectin was added uniformly on the working electrode to promote cell adhesion. The stable frequency value after one hour was taken as a reference value (∆)f = 0; before the addition of cells. The stable impedance value after one hour was considered as the initial impedance. Then 400 μl of medium, containing a specific concentration of cells, was evenly introduced on fibronectin surface in the culture chamber. Five different seeding densities: 500, 1.5 × 10^4^, 2.0 × 10^4^, 3.0 × 10^4^, and 5.0 × 10^4^ cells/cm^2^ were used for these experimental tests to validate the sensitivity of the device. The process of cell sedimentation at each seeding density was monitored repeatedly for three times and average magnitudes were taken to plot the final curve. The variations of the resonant frequency and impedance values were monitored for several hours to several days, during the entire process of cells attachment and spreading over the working electrode.

#### 4.2.1. Gravimetric Measurements with QCM Resonator

**Figure 8.**
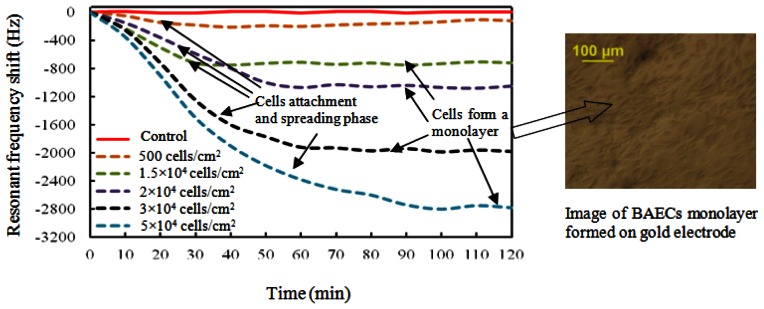
The average resonant frequency shifts along with time for 2 hours corresponding to cell sedimentation of different seeding densities of 500, 1.5 × 10^4^, 2 × 10^4^, 3 × 10^4^, and 5 × 10^4^ cells/cm^2^. The right picture shows the microscopic image of BAECs monolayer formed on gold electrode.

**Figure 9.**
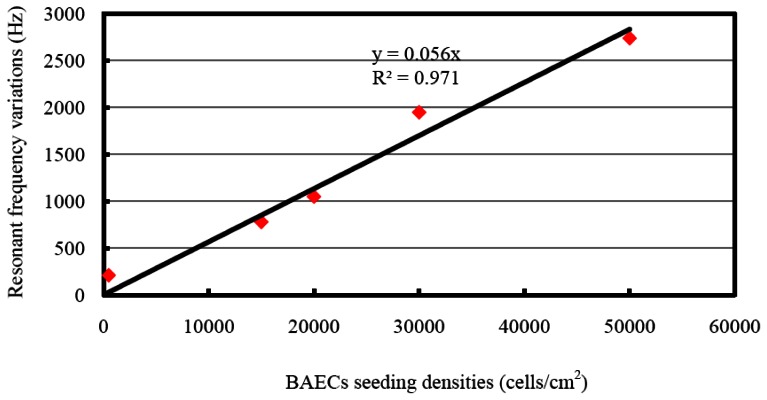
Linear relation between the resonant frequency shift and different BAECs seeding densities.

The resonant frequency shifts corresponding to different seeding densities are presented in Figure 8. The resonant frequency started to decrease at the inoculation of cells, and became more negative during the sedimentation of cells onto the top electrode. For seeding density of 1.5 × 10^4^ cells/cm^2^, the maximum frequency shift of 780 Hz was reached after about 35 minutes, followed by a saturated curve. The BAECs seeding density of 2.0 × 10^4^ cells/cm^2^ produced a resonant frequency shift of 1,050 Hz and needed about 60 minutes to form a compact monolayer on the hybrid sensor. The BAECs seeding density of 3.0 × 10^4^ cells/cm^2^ produced a frequency shift of 1,950 Hz and needed about 70 minutes for completing the process of attachment on the sensitive electrode. The highest seeding density of 5.0 × 10^4^ cells/cm^2^ took about 100 minutes to reach the maximum frequency shift of 2,740 Hz. The resonant frequency with BAECs seeding density of 500 cells/cm^2^ reached a maximum shift of 210 Hz initially, and then tended to increase. It was found by observation from microscope that the cells could not form a good monolayer at a seeding density of 500 cells/cm^2^. It is clear that higher cell seeding density produced larger frequency shifts. The resonant frequency of the control well remained constant during the whole experiment time span. The dependence of different BAECs seeding densities versus the changes of resonant frequency is shown in Figure 9. The graph indicates the negative resonant frequency shift increases with increasing number of BAECs attached on the electrode. The linear regression analysis has a significant value of R^2^ = 0.971, and indicates reasonable correlation between resonant frequency shifts and the number of BAECs. As reported in the literature the QCM resonator could be employed as ultrasensitive sensor for analyte detection in liquid [40]. This type of QCM had higher resonant frequency (39 MHz–110 MHz), higher sensitivity and lower linearity.

The Sauerbrey Equation (5), describes the relationship between the resonant frequency shift and the mass change as follows [41]:

(2) $$\Delta f=-\frac{2{f_{0}}^{2}\Delta m}{A\sqrt{\mu_{q}\rho_{q}}}=-C_{f}\Delta m$$

where ∆f is the resonant frequency shift; f_0_ is the resonant frequency of the quartz (16.52 MHz), ∆m is the mass change; A is the area of the top electrode (3.14 mm^2^); μ_q_ and ρ_q_ are the shear modulus (2.947 × 10^11^ g/cm.s^2^) and density (2.648 g/cm^3^) of the quartz substrate, respectively; C_f_ is the sensitivity. Based on the maximum resonant frequency shifts corresponding to different cell seeding densities, the change of mass on the sensitive working electrode was calculated using Equation (5). In comparison, finite element method using COMSOL Multiphysics™ was used to simulate the mass change of cell loading corresponding to different resonant frequency shifts. PMMA was used to simulate BAECs loaded on AT-cut quartz. The mass change was obtained and compared with that derived from Sauerbrey equation, as seen in Table 2. From Table 2, it can be seen that the mass change resulted from COMSOL simulation is less than the theoretical value. The reason is that the Sauerbrey equation is not valid for biological cells, which are considered as viscoelastic materials. More advanced model was created to study the mechanisms that are responsible for the frequency change in the case of biological materials with viscoelastic properties [12,42]. It was found that the frequency change is proportional to cell coverage, which depends on the seeding density before the coverage reaches 100%, and the cell-substrate gap and cell mechanical properties are similar for different seeding densities [12,42].

#### 4.2.2. Impedance Measurements and Discussion

Simultaneous gravimetric and impedance measurements were obtained using the hybrid biosensor when BAEC cells were added to the culturing chamber. The BAEC cells attached on the circular ECIS working electrode (also the upper acoustic wave excitation electrode for the QCM) and impedance measurements were performed from 40 Hz to 100 kHz, between the ECIS working and counter electrodes using an impedance analyzer. The greatest discrepancy of impedance measurements between control wells containing only media and wells containing cells and media was found at 40 kHz. Therefore, 40 kHz was used as optimal frequency for all the impedance measurements.

**Figure 10.**
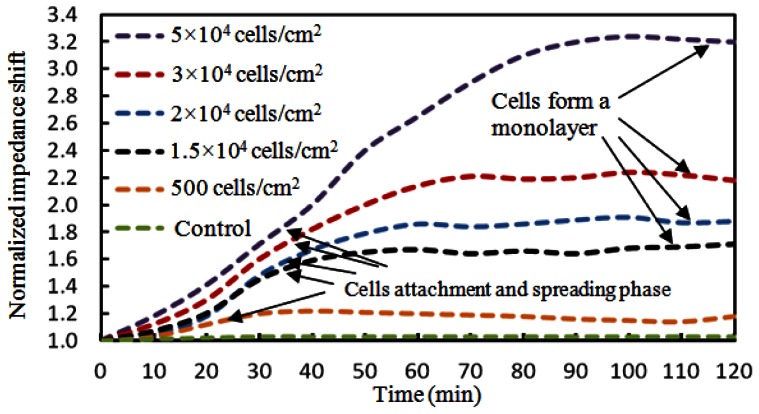
The normalized impedance change along with time for 2 hours corresponding to cell sedimentation of different seeding densities of 500, 1.5 × 10^4^, 2 × 10^4^, 3 × 10^4^, and 5 × 10^4^ cells/cm^2^ at 40 kHz.

**Figure 11.**
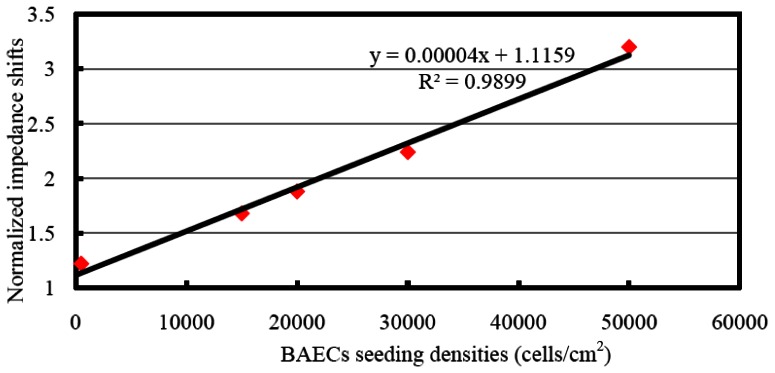
Linear relation between normalized impedance values coresponding to and different BAECs seeding densities.

The BAEC cells attached on the ECIS working electrode act as insulators and the impedance values increase during the attachment process. As BAEC cells grow and cover the electrodes, the current is impeded in a manner related to the number of cells covering the electrode, the morphology of the cells and the nature of the cell attachment. When the BAECs form a compact monolayer the impedance values are constant. The graphs in Figure 10 show the attachment and spreading behaviours of five different concentrations of BAEC cells: 500, 1.5 × 10^4^, 2.0 × 10^4^, 3.0 × 10^4^, and 5.0 × 10^4^ cells/cm^2^. According to Figure 9, the BAEC cells with seeding density of 1.5 × 10^4^ cells/cm^2^ reach confluence in about 45 minutes. Upon reaching confluence the cells have constant impedance values for long time. The BAECs with higher impedance reach confluence in 60, 70 and 100 minutes, respectively. The impedance with BAECs seeding density of 500 cells/cm^2^ increased to a maximum in about 20 minutes and then tended to decline instead of remaining saturated as that with other higher seeding densities. The reason is believed to be that the number of BAECs is too small to form a monolayer. The impedance value of the control well remained constant during the whole experiment. The dependence between the BAECs seeding density versus the change in normalized impedance values is shown in Figure 11. The normalized impedance values were obtained as the ratio between the impedance values of cells membrane and the impedance values of media without cells from the control well. The graph indicates the normalized impedance shift increases with increasing number of BAECs attached on the electrode, see Figure 11. The linear regression has a significant valuers of R^2^ = 0.989, and indicates reasonable correlation between normalized impedance shifts and the number of BAECs.

These gravimetric and impedimetric measurements on the same cell monolayer demonstrate that the hybrid biosensor is able to perform two different types of measurements at the same time and this sensor could be successfully tested in the future with water containing toxicants. The experimental measurements described by Figures 8 and 10 showed consistent information and the cell attachment and spreading could be monitored in an accurate manner with both sensors on a single chip.

## 5. Conclusions

The hybrid biosensor presented in this paper combines two biosensing techniques; resonant frequency measurements and ECIS in one device. The biosensor is able to simultaneously perform in real-time two different types of electric measurements on the same BAECs cell monolayer: (1) monitoring the resonant frequency of the QCM resonator that will give information about the progression of cells adhesion, viability and quantity of the attached cells and, (2) recording the impedance spectra of the cells, that contains information on cell attachment and viability.

In order to combine the two measurement techniques on a single chip the working electrode for ECIS technique acts as the top electrode of the QCM resonator. In this way, the top metal electrode used for generating the acoustic wave is also used for ECIS measurements of live cells.

This novel hybrid biosensor was cultured with live cells. The BAECs were used for device testing, because of their ability for long-term survival on the hybrid biosensor in a laboratory environment.

Several BAEC densities were used for gravimetric and impedimetric measurements performed over a period of time. The time necessary for the cells to attach and form a compact monolayer is the same in the case of gravimetric and impedimetric measurements. The time necessary for the BAEC cells to attach and form a compact monolayer on the biosensor is 35∼45 minutes for 1.5 × 10^4^ cells/cm^2^ BAECs; 60 minutes for 2.0 × 10^4^ cells/cm^2^ BAECs; 70 minutes for 3.0 × 10^4^ cells/cm^2^ BAECs; and 100 minutes for 5.0 × 10^4^ cells/cm^2^ BAECs. The lowest cell density seeded on the sensor was 500 cells/cm^2^ and the corresponding recorded resonant frequency shift was 100 Hz. The highest cell concentration used in this experiment was 5.0 × 10^4^ cells/cm^2^ and produced a frequency variation of 2,800 Hz. From the calibration curve (Figure 9) it can be deduced that the saturation values at this high cell density were still not reached. We did not used a higher density of cells because cells at high density will not have enough space to attach to the substrate and some cells will be forced to grow on top of other cells or will float in the media and will be pushed out when the media is changed. Also a very low concentration of cells requires long time to form a compact monolayer. In this paper it was demonstrated that the BAECs can be cultured on the multiparametric sensor and precise electric measurements could be recorded. Cell densities of 1.5 × 10^4^ cells/cm^2^ or 2.0 × 10^4^ cells/cm^2^ are best suited for this application.

In the future this hybrid biosensor will be employed for testing water containing toxicants. Cell damages induced by chemicals from the water sample will be observed as a decrease of impedance values, as well as an increase in the resonant frequency. When tested with toxic water, this original multiparametric sensor system is expected to decrease the false positive rate and increase the accuracy of the detection, since responses from two sensors will be considered.

## Figures and Tables

**Table 1.** The material properties of AT-cut quartz crystal in simulation.

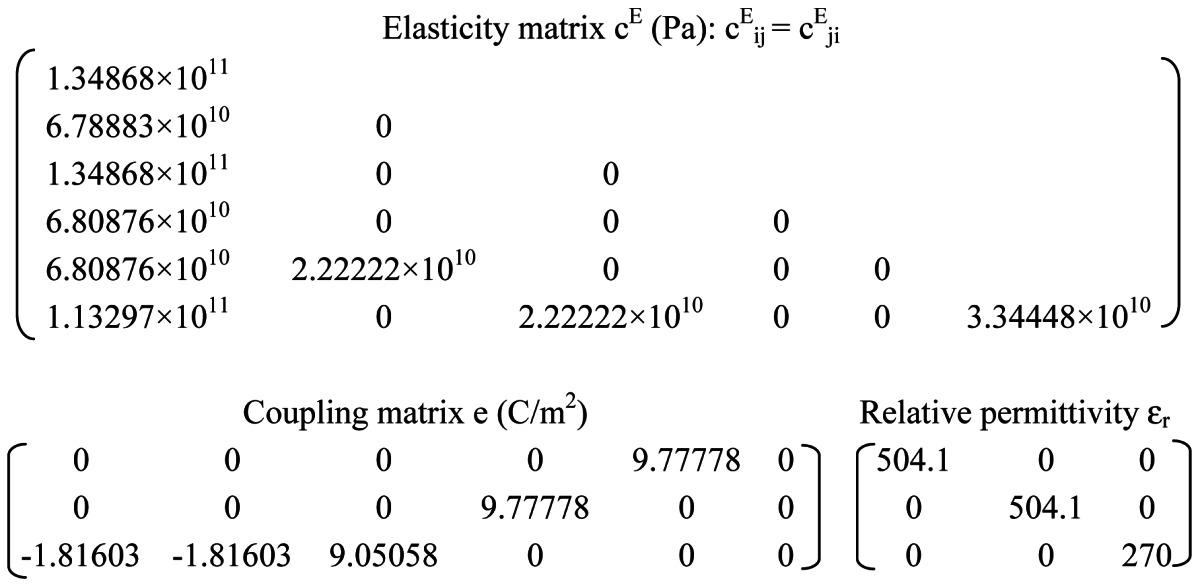

**Table 2.** The mass change magnitude corresponding to different frequency shifts by using Sauerbrey Equation (5) and COMSOL simulation.

**Cell Seeding Density (cell #/ cm^2^)**	**500**	**15,000**	**20,000**	**30,000**	**50,000**
Frequency change Δf (Hz)	210	780	1,050	1,950	2,740
Theoretical mass change Δm (ng)	11	40	53	99	138
COMSOL simulation Δm (ng)	10	38	49	91	131
